# Somatic Outcomes and Nutritional Management of Anorexia Nervosa

**DOI:** 10.3390/nu15112541

**Published:** 2023-05-30

**Authors:** Chantal Stheneur, Mouna Hanachi

**Affiliations:** 1 Fondation Santé des Etudiants de France, University Center for Adolescent and Young Adult Health, 75014 Paris, France; 2 CESP, INSERM, UMR 1018, University Paris-Sud, 94800 Villejuif, France; 3 Simone Veil Faculty of Medicine, UVSQ, Paris-Saclay University, 78690 Saint-Quentin en Yvelines, France; 4 Clinical Nutrition Unit, Paul Brousse University Hospital, Assistance Publique-Hôpitaux de Paris, 94800 Villejuif, France; mouna.hanachi@aphp.fr; 5 INRAE, AgroParisTech, Micalis Institute, Paris-Saclay University, 78354 Jouy-en-Josas, France

This Special Issue of *Nutrients*, entitled “Nutritional Management and Outcomes in Anorexia Nervosa”, aims to advance aspects of the scientific understanding of some serious or frequent somatic involvements and of the precocious nutrition management of severe forms of the disease in order to assist clinicians in the management of such patients. This publication compiles a variety different articles on the management of nutrition, with applications and topics of discussion ranging from the emergency of extreme undernutrition to a detailed evaluation of energy expenditure to adapt outpatient care as closely as possible. We also furnish studies on non-nutritional management, such as support for physical exercise or management of bone demineralization.

Anorexia nervosa (AN) is a severe eating disorder that affect 1.4% of women and 0.2% of men [1]. It is the most lethal psychiatric disorder, with a standardized mortality rate of 5.6, and rising up to 15.9 for extremely severe patients [2]. The two principal mortality causes are somatic complications and suicide. AN is defined by refusal and fear to maintain a normal body weight coupled with a severe disturbed body image [3] and restricted calorie intakes. It has two distinctive sub-types: the pure restricting sub-type (AN-R) and the binge-eating/purging sub-type (AN-BP). The latter combines, in addition to restriction, binge eating and purging actions (vomiting and/or laxative abuse). Understanding these subtypes is essential for the accurate diagnosis and effective treatment of anorexia nervosa.

Food restriction and semi-prolonged fasting can lead to marasmus, a protein-energetic adaptative malnutrition that constitutes the primary form of malnutrition found in AN. However, the acute hypercatabolic type of the condition can be observed in some cases. This form combines a lack of intake and metabolic stress, resulting in protein loss, loss of body mass and loss of protein, muscle mass and function. The translation of metabolic stress is mainly biological (inflammatory syndrome and/or decreased albumin), but different clinical features could be included such as peripheral edema, hypoalbuminemia, fatty liver, skin and hair lesions. Individuals with the binge-eating/purging subtype of AN may have a higher risk of medical complications, such as electrolyte imbalances or gastrointestinal problems, while those with the restricting subtype may experience more severe malnutrition and organ damage. Severe undernutrition and pathological behaviors have the potential to lead to vital organ failure such as heart, liver and less known kidneys. Heart complications include cardiovascular dysfunction, heart failure, arrhythmia, and sudden cardiac death. Liver abnormalities are also frequent, and sometimes life-threatening, in undernourished AN patients. In adult patients, hypertransaminemia is found in almost half of the cases. Two types of hypertransaminasemia can be distinguished: (A) undernutrition hypertransaminasemia related to the importance of undernutrition. It is frequently moderate, with spontaneous favorable evolution. Its physiopathology involves starvation-induced autophagy and glycogen hepatocyte depletion. (B) The second variety is renutrition hypertransaminasemia, which is most often moderate and rarely exceeds values higher than 10 times the norm. More recently, renal function has been reported as showing alteration in patients with AN. Over 70% of people suffering from AN may experience renal complications at some point in their lives, with a 5.2% prevalence of severe kidney disease, including terminal renal failure, after 21 years of AN [4].

In addition to these life-threatening complications, others had significant impacts on the quality of life. Constipation is among the most commonly reported symptoms in the literature on patients with eating disorders. The link with the framework of irritable bowel syndrome is more and more often reported. Its prevalence may be higher than 50%. The evolution is moreover independent of that of the AN, with a high risk of persistence even after the recovery from AN. Among other complications, we cite amenorrhea, decreases in bone mineral density and fractures as also being extremely frequent. In patients presenting purging behaviors, especially vomiting, dental injuries are very frequent and must be researched carefully. The damage can be done to all the components of the mouth such as the dental enamel, the gingiva, and any other area, which can lead to a major functional and aesthetic impact. It is essential to recognize these alterations at an early stage in order to be able to treat them before the definitive sequelae. In addition to local treatment, hygienic measures, as well as medications such as proton pump inhibitors, can be recommended.

Problematic physical activity may be associated with AN and leads to more frequent and severe somatic complications. Indeed, some patients tend to engage in different forms of physical exercise, regardless of their malnourished state and the risks associated, aggravating malnutrition and prognosis. Fatigue fractures and skin lesions due to friction are both common consequences of this practice. However, physical activity, if undertaken in the proper fashion, can also help in the management of the condition and return to a better state of health.

To limit all these complications, weight gain is needed. Treatment begins with the stabilization of the somatic and nutritional state before psychiatric care can begin. In cases of severe malnutrition, nutrition must start with enteral nutrition (EN). This is indicated if the undernutrition is severe (BMI < 13 in adults), and/or associated with metabolic disorders and/or if prolonged weight stagnation despite adequate nutritional and psychiatric management [2]. Renutrition must be monitored to avoid the development of refeeding syndrome while promoting rapid weight gain. Whether in meal feeding or in EN, calories should be increased gradually in the first days. The renutrition by EN must last for a limited time and natural feeding must be encouraged to allow the maintenance of eating habits. This oral feeding should be monitored by an experienced dietician to help the patient to gradually increase their intake and diversify their diet. Parenteral nutrition is contraindicated in AN because of the high risk of metabolic and infectious complications. If the vital risk is no longer engaged after a few days of renutrition, the diet must continue to be monitored in order to gradually restore intakes in a way that is adapted to the needs of the patient and includes all necessary nutrients.
